# Some Medical Observations in Europe

**Published:** 1898-12

**Authors:** J. J. Mulheron

**Affiliations:** Detroit, Mich.


### SOME MEDICAL OBSERVATIONS IN EUROPE.

BY J. J. MULHERON, M.D., DETROIT, MICH.

There probably lurks or has lurked in the breast of nearly every American physician a desire to round out his medical studies here with a postgradute course at one of the European medical centers. This being conceded, the question of when and where to go presents itself, and as I have recently returned from a six-months' tour embracing these centers, my observations may not be wholly uninteresting.

First, then, at what period in the American graduate's career is he likely to find his European visit most profitable? Many, thanks to a patrimony, take it immediately after their graduation, and many, too many, are obliged, through the chronic discrepancy between receipts and disbursements, to defer it until they have entered the ranks of the silver-grays. In my humble opinion neither of these periods is adapted to the fullest benefit derivable from such a visit. This fullest benefit resides chiefly in the education of the senses in connection with diagnostic examinations. Clinical teaching to nndergraduates in this country has up to the present time consisted in the teacher's telling his class what his senses have revealed during the course of his examination of the patient. In no country are young men better grounded than are the graduates of a number of our medical schools in the significance of physical signs and subjective symptoms. In the matter of the teaching of our young men through the education of their senses—seeing, hearing, feeling, and even smelling and tasting—how to discover such signs and symptoms, I believe I can say it advisedly, our country is sadly in the rear. The fullest benefit to be derived from a postgraduate European course lies in its supplementing theoretical instruction with that education which comes from actual bedside contact with the patient under the direction of master diagnosticians. The American Indian traces his victim over trackless expanses by means of foot-prints and broken twigs and distorted blades of grass, which the acutest visioned white, not specially trained in this direction, can not detect. And no one who has visited the clinics of Europe—those of Vienna, for example—can fail to have been struck by the acute perceptive powers of the gentlemen whose lives have been devoted to the detection of the aberrations from the physiological standard.

The training of the perceptive faculties is of course best accomplished during the plastic period of youth, and if there were no other considerations one would naturally urge the young man to take the European postgraduate course as soon after his graduation here as possible. But there are other considerations. The requisites of the successful practice of medicine are not all to be acquired in the class-room. For instance, that easy deportment at the bedside, which carries the impression that you are master of the situation, the ability to maintain one's mental equilibrium in the face of unfavorable or even positively adverse influences, the power of mental abstraction or concentration in summing up the evidences which determines the diagnosis, the discovery of one's special aptitudes in certain directions—these qualifications come to the average physician during the first few, say two or three, years of practice, although in individual instances they come later, and in some they never come at all. It is well to have acquired them before taking the foreign course. The work will then be more satisfactory in that it will be more apt to be directed along the lines of best advantage. The few years of interregnum between undergraduate and systematic postgraduate work will also be an advantage. At its close the young man will start in again with renewed vigor and enthusiasm.

The danger in postponing the trip to the autumnal days of life lies chiefly in the facts that the theoretical knowledge so necessary as a basis for advanced practical work is then apt to have escaped, and that scientific enthusiasm is prone to be on the wane. These things are among the infirmities of age. Age is, however, only a relative thing. Some men are old at fifty and some are young at seventy. Physically, as has been truly said, a man is just as old as his arteries. We have no such convenient gauge of a man's mental age, but we know that mental activity may be prolonged quite indefinitely under the stimulus of the genuine scientific enthusiasm engendered in youth. Science, though a jealous mistress, is likely to remain steadfast and leal to those who court her early. Lord Lansdowne, at the age of eighty, has commenced the study of Arabic.

It will quite naturally be asked what advantage Europe presents over this country in the matter of postgraduate medical instruction. While I have some acquaintance with the advantages offered in a number of the cities of the two hemispheres, I am better prepared to speak by the card of those of New York and Vienna, having taken a postgraduate course in each. These two are fairly representative of their respective sides of the Atlantic. In the first place, the material at Vienna is much more concentrated, owing to the smaller number of hospitals. The loss of time in traveling from hospital to hospital, miles apart, is thus obviated. The Allgemeine Krankenhaus in Vienna, with its upwards of 3,000 beds, affords under its roof as much material as the half-dozen largest and widely separated hospitals of New York. One may enter this immense hospital at seven in the morning and be continually occupied until seven at night—without the loss of an hour's time in going to and fro among the clinics. The advantage of this is very apparent.

The concentration of this large amount of material under the one management renders possible the selection of a practically unlimited quantity under each of the different divisions in which the student may be interested.

The control of the material at Vienna is, moreover, to a degree which public sentiment would not tolerate in this country. The patient entering the Allgemeine Krankenhaus surrenders himself absolutely to the authority of the institution. The people, too, are more docile than those who have tasted the sweets of our American personal and political liberty. During their life in the Allgemeine Krankenhaus they are clinical material and are so treated, devoid of all sentimentality. One is struck with the absence of the amenities which the American physician observes in his handling of even his pauper patients, whom he is expected to handle as delicately and with as much regard for their feelings as if they were of his best clientele.

After death this clinical material in the Allgemiene Krankenhaus becomes pathological material for study and investigation. Regardless of sex, social condition, or any other consideration, every patient dying in this institution is subjected to an autopsy. The decedent is sent to the dead-room with a pewter tag bearing his number tied to his toe. The diagnosis numbered to correspond with the tag accompanies him and is filed on a spindle where it is open to inspection. The autopsy is no mere examination of the seat of the disease as stated in the report of the diagnostician. It is made regardless of this report. The pathologist starts in at the head and literally dissects the subject to the very feet. He then files his report. I have seen as many us fifteen bodies thus examined in the course of a forenoon. Sections of the pathological tissue are removed for microscopical examination. This close tab kept on the diagnostician by the pathologist is intensely stimulating to the former. It is an effectual safeguard against careless or slipshod diagnosis and treatment based on subjective symptoms. It is exceedingly interesting to watch the attending physician as he notes the revelations made by the pathologist's knife in an obscure case. His reputation as well as his bread and butter are at stake, for discrepancies too frequent or too glaring between his report and the pathologist's findings have the result of bringing him on the carpet. I can imagine no situation so well calculated as this to stimulate keen scientific interest. All directly concerned are, moreover, through personal as well as scientific motives, kept constantly on edge, and the interest aroused by the operation of the system is certainly contagious and never lags.

I would make no invidious comparison between the teaching faculties of the two countries. There is this, however, to emphasize of the European teacher, and particularly as I was familiar with him in Vienna: He is, as a rule, a devotee of his calling, gauging his success not so much by emolument as by scientific results. His wants are not extravagant, and his salary, while not large, is usually sufficient to cover his desires. There is in the profession much less of the feverish lust for money that is noticeable in this country. The fact is perhaps largely due to the general hopelessness of pecuniary ambition, for the profession is miserably paid abroad.

The essential postgraduate teaching is chiefly done by the assistants to the professors. The latter are, as a rule, occupied with their private clienteles to the exclusion of other than didactic instruction and a general supervision of the clinical work. Such teaching as they do is very much of the nature of that done by clinical teachers in this country. Some have therefore visited merely the public clinics abroad and have seen nothing in them superior to what we have at home. To secure the clinical teaching which I have referred to as the education of the senses in the detection of the pathological conditions, it is necessary to take the "Curses." I took the "Curses" in internal medicine, gynecology, and obstetrics. The "Curse" consists essentially of a limited number of students making systematic clinical examinations under the direction of the clinician in charge of the material presented. That on internal medicine was conducted by Mannaberg, first assistant to Nothnagel. The class here was restricted to ten, and these had the run of three wards containing upwards of one hundred beds. Each member of the class is assigned two patients each day. These are considered peculiarly his, and he is required to secure and record their anamneses and such points in their family history as bear on the case; this in addition to the most exhaustive physical examination by palpation, percussion, auscultation, analysis of excreta, etc., with a view to diagnosis. He is allowed unlimited time, but when his case is selected for demonstration it is very seldom that he feels that he has spent any unnecessary time in investigating. He is not confined to these two cases, but has all the patients in the ward at his disposal. He is, however, not expected to stand an examination on others than those assigned him. The examination is in connection with the cases demonstrated, and in addition to being a thorough test of the student's ability to discover the signs and symptoms, is highly instructive in the methods employed in the institution. The thoroughness of the examination and the attention to the minutest details are a revelation to one who has noted the methods of diagnosing in vogue elsewhere. Not only are the lines indicated in the anamnesis followed up, but the investigation, except in the most palpable cases, leaves no organ of the body untouched. The examination is not alone in the interest of the patient, but also in that of science, and, indeed, one is often made to feel that the interests of the latter are paramount.

When the patient has been given the benefit of an exact diagnosis, interest in him seems to have declined. Therapeutics is little discussed. The scientific faith in the *vis medicatrix naturæ* has developed a condition approaching therapeutic nihilism. Therapeutics is regarded as too inexact a science (if science it can be called) to very seriously engage the attention of these scientific minds. They are sadly in the rear in the matter of the practical therapeutics which gives the American physician his great advantage in competition with the European graduate. So intent is the latter on prescribing only for pathological conditions which have been revealed to his senses, that he is prone to regard the relief of symptoms as an obstacle to correct diagnosis, and therefore unscientific and not to be countenanced. To give an antipyretic, for instance, to simply reduce the temperature, which may be an important symptom in arriving at an exact diagnosis, would not be tolerated. The cause of the increased heat must be discovered and treated. That such treatment is the more scientific can not, of course, be gainsaid, but that it is more successful in general practice is open to question. Of the fastidiousness of the patient's palate the European hospital physician takes little or no account, and fortunately for his practice his patients do not look for any of the refinements which American pharmacy has placed in the hands of the profession of this country.

My "Curses" in gynecology were taken at the clinics of Professors Chrobak and Schauta respectively, and under the tutelage of assistants Knauer and Schmidt. Of these, and especially of that of Knauer, it would be difficult to speak in terms of exaggerated praise. The work was, necessarily, principally diagnostic. Between these two clinics three hours were spent each day in making digital examinations. The variety of material selected from the clinics covered quite completely the whole list of the ailments to which woman is heir. Six students were received in each "Curse," and the number of patients allotted to each averaged fifteen per day. All the cases to be operated on the following day were, in addition, examined. Knauer commenced to operate at 8 a. m. and was busy until 10. His principal, the genial Chrobak, seldom operated in the "Curse," his work being confined almost exclusively to his public clinic. Knauer is a man of about thirty-five, and although other gynecologists abroad have been able, through combinations of circumstances, to become more noted, I hazard nothing in saying that none of them are his superiors in all the essential qualifications in this special line of surgery. Marvelously correct in his diagnosis of adominal tumors, he is also a master of the operative technique. His immediate assistants and the members of his "Curse" are alone admitted to his operating-room. It is needless to say that the perfection of asepsis is aimed at in his operation. The absolute control of the clinic which is permitted him by Chrobak is the highest tribute which could be paid any man. He is moreover a charming gentleman, and I shall ever cherish my recollection of the few months I spent with him. Schmidt, first gynecological assistant to Schauta, is also a gynecologist of much promise. He is unfortunate in being subordinate to a man of Schauta's peculiarities. While this gentleman is unquestionably a gynecologist of the first rank, his personality is not attractive, and such is his apparent desire to be "the whole thing" that his assistants are afforded limited opportunity to develop themselves. While the atmosphere which surrounds Chrobak is sweet and redolent of *bonhomie*, that which pervades Schauta's presence is acid and dispiriting.

It was my privilege also to accept kind invitations to Wertheim's operations at the Bellina-pavilion of the Kaiserin Elizabeth-Spital. This pavilion was built by the resident member of the Rothschilds family in Vienna. It contains sixty beds and cost half a million florins ($200,000). It is probably the most completely and scientifically equipped gynecological institution extant, and certainly gynecology has nowhere a more scientific exponent than Wertheim. As an operator he is what might be termed "brilliant" alike as regards technique, boldness of conception and promptness of action in the exigencies developing during an operation. The operation of shortening the round ligaments through the vagina for the retention of the uterus in normal anteversion is of his devising. It was my pleasure to witness his performance of it a number of times, as well as to see it done several times by Knauer and Schmidt at the Allgemeine Krankenhaus. It is apparently the ideal operation for retroversion and devoid of the danger necessarily attendant on laparotomy.

In the field of obstetrics Vienna stands undisputedly in the lead. There are three obstetrical clinics at the Allgemeine Krankenhaus, under the control, respectively, of Schauta, Chrobak, and Braun, the material being evenly divided among the three. The number of confinements is upwards of 11,000 annually. And not alone does this immense quantity of material give Vienna pre-eminence; the nature of the material is also different from that found elsewhere. The underfed condition of the poorer classes in Austria-Hungary and the bad sanitary conditions under which they are reared have developed an alarming prevalence of rachitis or "die Englische Krankheit," as it is for some unaccountable reason called. The result is that nearly twenty-five per cent. of the lying-in patients have contracted pelves. The frequency of instrumental interference can thus be conjectured. During my attendance of two months at Schauta's obstetrical clinic, under his assistant Neumann, I witnessed no less than five craniotomies. This procedure, to be followed later on by vaginal extirpation of the uterus, has taken precedence there over the Porro operation. It is held to be much more favorable in its results, which are measured by the degree of safety to the mother. The possibility of saving the child through the Porro operation is not great enough to warrant the increased jeopardy in which the mother's life is placed.

The aseptic precaution in the obstetrical clinics are quite as stringent as those at the surgical clinics. I must reserve any statement of the details, as well as the narration of my experiences at other places than Vienna, for another occasion.—*Medical Age.*

